# Management of fat embolism co-existing with thromboembolism may be challenging!

**DOI:** 10.4103/2321-3868.142396

**Published:** 2014-10-25

**Authors:** Kapil Dev Soni, Richa Aggarwal, Gopal Jalwal

**Affiliations:** 1 Department of Critical and Intensive Care, Jai Prakash Narayan Apex Trauma Centre, All India Institute of Medical Sciences, New Delhi, 110029 India; 2 Department of Anesthesia, Jai Prakash Narayan Apex Trauma Centre, All India Institute of Medical Sciences, New Delhi, India

Dear Editor,

Fat embolism syndrome (FES) co-existing with venous thromboembolism is rare entity. It is a serious clinical disorder occurring after trauma, orthopedic procedures and rarely in non-traumatic patients.[1,2] We report one such case, which posed clinical dilemma in the management. A 20-year-old male patient was admitted to the hospital with bilateral closed fracture of the femur and bilateral both bone leg fractures following a road traffic injury. He was referred to our Center approximately 36 hours after the accident. On admission he was fully awake, obeying commands and hemodynamically stable. Electrocardiogram showed S1Q3T3 pattern, suggestive of pulmonary embolism. He was intubated and shifted for computed tomographic pulmonary angiography (CTPA) that showed small thrombus in segmental branch of right pulmonary artery. He was put on heparin infusion and transferred to Intensive Care Unit (ICU). In ICU, he was placed on Volume Control Ventilation mode with 100% FiO_2_. He was hemodynamically stable (BP-140/76 mm Hg) with tachycardia (pulse rate-140 beats/min). Three hours later, the patient became irritable, confused with a drop in sensorium. His oxygen saturation fell to 80% from the baseline value of 98%, increasing to 90% on 100% FiO_2_ with recruitment maneuver. The chest X-ray was normal. Arterial blood gas analysis showed PaO_2_ of 62 mmHg on 100% FiO_2_. By now the patient developed petechial rash all over his upper trunk including axillae [Figure 1]. Ophthalmic fundoscopy revealed retinal hemorrhages. Fat embolism was suspected based on clinical findings. Low tidal volume with high peep ventilation strategy was continued to maintain SpO_2_ within acceptable limits. Two days later, the patient’s chest X-ray showed diffuse fluffy infiltrate with PaO_2_/FiO_2_ ratio of 70. Blood investigations showed low platelet counts (70 × 10^3^/µL) and hemoglobin (8.1%). CT head indicated features of cerebral edema. These findings further confirmed the diagnosis. Heparin Infusion was stopped. The patient’s condition improved remarkably over the next few days. He was tracheostomised early in the course of illness, later decannulated and 25 days later discharged from the ICU in healthy clinical condition.

**Table 1:** 

Access this article online	
**Quick Response Code**:	**Website**: www.burnstrauma.com
	**DOI**: 10.4103/2321-3868.142396

**Figure 1:**
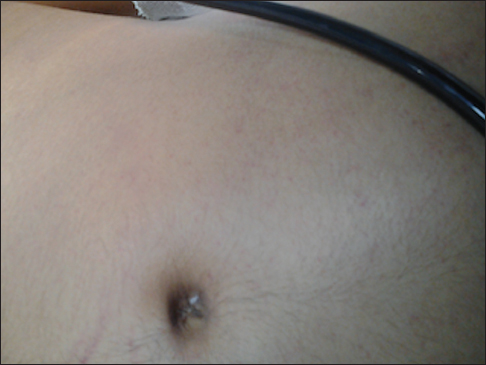
Showing characteristic petechial rash of fat embolism over the chest and anterior abdomen.

## Discussion

Fat emboli typically manifest 24–72 h after initial insult. Affected patients presented with the classic triad of hypoxemia, neurological abnormality and petechial rash.[1,2] Fat embolism could be difficult to diagnose. There is no diagnostic test that is sensitive or specific for confirmation or exclusion. Imaging studies are most useful in diagnosis of FES. X-ray chest radiograph often shows increased pulmonary markings and flake like pulmonary shadow (snowstorm appearance).[3] CT study is insensitive while magnetic resonance imaging is the most sensitive technique in demonstrating cerebral changes of FES.

Pathology of FES is incompletely understood. FES might be due to direct effect of fat globules entering into the blood stream following tissue damage or alternatively production of fat intermediaries such as chylomicrons or lipids may play a role.

Treatment of fat embolism is only supportive.[4,5] Our case is unique as our patient developed FES in presence of venous thromboembolism diagnosed on CTPA. The management of FES becomes challenging due to the controversial role of heparin in potentiation of FES pathogenesis. Heparin activates lipase which increase free fatty acid and augment fat emboli production. Therefore, heparin was stopped as FES was more severe in comparison to peripheral segmental thrombi in pulmonary artery. Once the patient recovered from the FES, heparin was restarted for the existing pulmonary embolism.

## New Organization on Burn Injury Established!

### The International Association of Burn Injury (IABI)

The IABI is a nonprofit public academic organization, which intent to supply a platform for burn surgeons to exchange information and knowledge on the treatment and research of burn injury in different regions and countries. The goal of IABI is to fuel the progress of treatment and research of burn injury and improve the recovery rate of burn patients in the world. Standardization the diagnosis, treatment and prevention of burn injury and its complications is another task of IABI.

The IABI has three standing sections; they are academic committee, administrational office and Li Ao Burns Surgery Foundation. About 20 members constitue the standing academic committee, which includes one chairman and 2–3 vice chairmen, one general secretary. The academic committee is responsible for decision and direction of convoking the IABI congress every three years, holding aperiodic academic program, encouraging exchanging and advanced study of burn surgeons, and recommending the candidate owners of the Li Ao Burns Surgery Award.

### The Chinese Burn Care and Rehabilitation Association (CBCRA)

The founding ceremony of Chinese Burn Care and Rehabilitation Association was held on October 10, 2014 in Chongqing, China. Eighty-six members from 24 provinces attended the founding ceremony. The Chinese Burn Care and Rehabilitation Association focuses on improving the life quality of burn patients, facilitating communication and collaboration between burn care and rehabilitation specialists all over the world.
